# Expression and biological function of the cellular communication network factor 5 (CCN5) in primary liver cells

**DOI:** 10.1007/s12079-023-00757-8

**Published:** 2023-05-11

**Authors:** Erawan Borkham-Kamphorst, Steffen K. Meurer, Ralf Weiskirchen

**Affiliations:** Institute of Molecular Pathobiochemistry, Experimental Gene Therapy and Clinical Chemistry (IFMPEGKC), RWTH University Hospital Aachen, Pauwelsstr. 30, 52074 Aachen, Germany

**Keywords:** Portal myofibroblasts, Endoplasmic reticulum stress, Unfolded protein response, Bile duct ligation, Hepatic stellate cells, Liver fibrosis, TGF-β1

## Abstract

The cellular (centralized) communication network (CCN) factor protein family contains six small secreted cysteine-rich proteins sharing high structural similarity. These matricellular proteins have vital biological functions in cell adhesion, migration, cell cycle progression, and control of production and degradation of extracellular matrix. However, in liver the biological functions of CCN proteins become most visible during hepatic injury, disease, and remodeling. In particular, most of the hepatic functions of CCN proteins were derived from CCN2/CTGF, which becomes highly expressed in damaged hepatocytes and acts as a profibrogenic molecule. On the contrary, CCN1/CYR61 seems to have opposite effects, while the biological activity during hepatic fibrosis is somewhat controversially discussed for other CCN family members. In the present study, we analyzed the expression of CCN5/WISP2 in cultures of different types of primary liver cells and in an experimental model of hepatic fibrosis. We found that CCN5 is expressed in hepatic stellate cells, myofibroblasts and portal myofibroblasts, while CCN5 expression is virtually absent in hepatocytes. During hepatic fibrogenesis, CCN5 is significantly upregulated. Overexpression of CCN5 in portal myofibroblasts reduced expression of transforming growth factor-β receptor I (ALK5) and concomitant Smad2 activation, whereas JunB expression is upregulated. Moreover, elevated expression of CCN5 induces endoplasmic reticulum stress, unfolded protein response and apoptosis in portal myofibroblasts. We suggest that upregulated expression of CCN5 might be an intrinsic control mechanism that counteracts overshooting fibrotic responses in profibrogenic liver cells.

**Graphical abstract:**

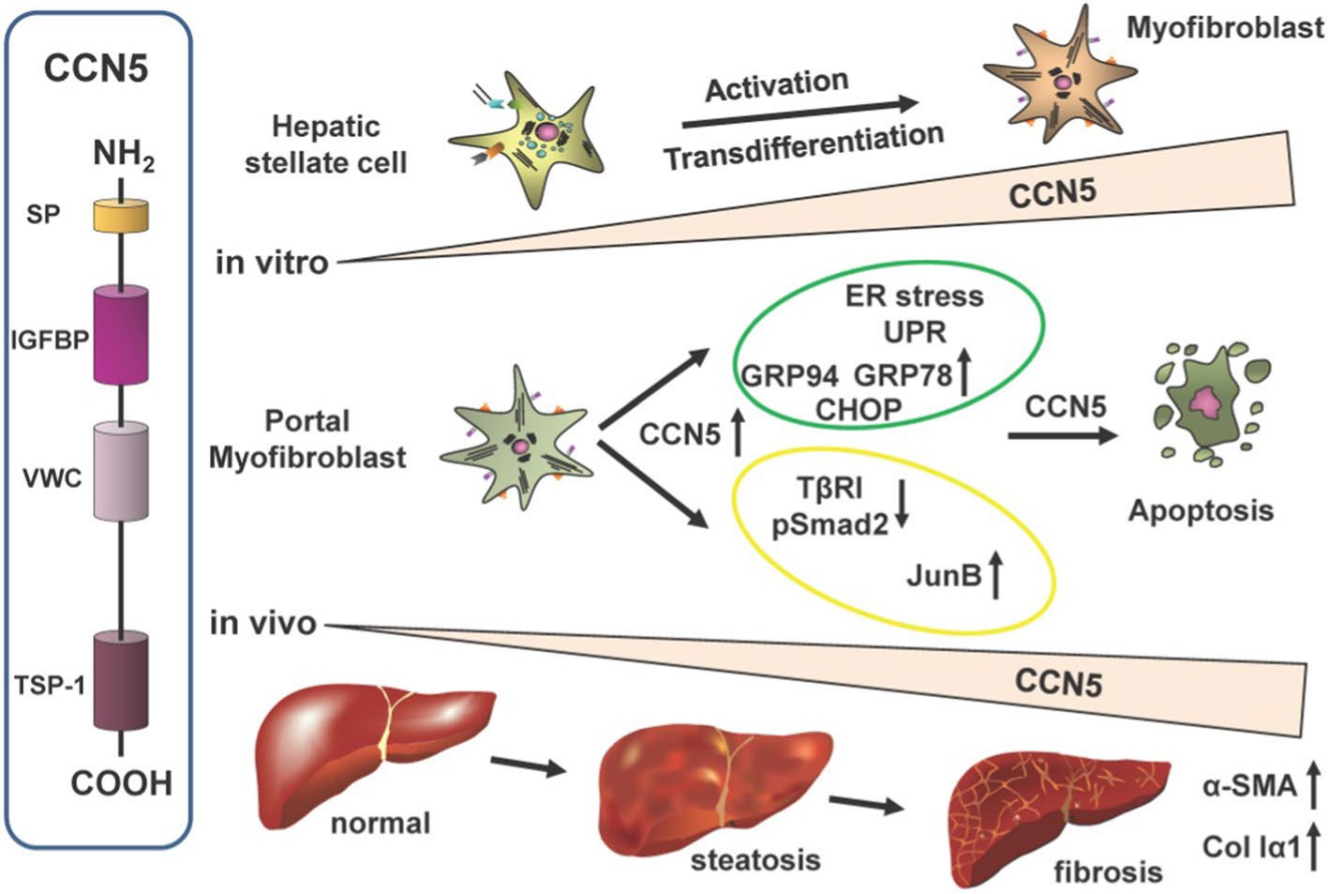

**Supplementary Information:**

The online version contains supplementary material available at 10.1007/s12079-023-00757-8.

## Introduction

The cellular (centralized) communication network (CCN) factor gene family includes six homologous members, formerly known as cysteine-rich angiogenic inducer 61 (CYR61/CCN1, OMIM: 602369), connective tissue growth factor (CTGF/CCN2, OMIM: 121009), nephroblastoma-overexpressed gene (NOV/CCN3, OMIM: 164958), Wingless-type MMTV integration site family, member 1 (WNT1)-inducible signaling pathway 1 (WISP1/CCN4, OMIM: 603398), WNT1-inducible signaling pathway 2 (WISP2/CCN5, OMIM: 603399), and WNT1-inducible signaling pathway 3 (WISP3/CCN6, OMIM: 603400) (Perbal 2018). These cysteine-rich proteins contain an *N*-terminal signal sequence and up to four individual structural modules including an insulin-like growth factor binding domain, a von Willebrand factor type C motif, a thrombospondin type I module and a carboxyl-terminal cysteine knot (Weiskirchen 2011). CCN5 lacks the C-terminal cysteine knot (CT)-domain, which governs amongst other functions homo- and heteromeric protein interactions, as well as binding to cytokines and receptors (Holbourn et al. 2008; Russo and Castellot 2010). The soluble CCN proteins are secreted extracellular matrix (ECM)-associated proteins. Thereby, they are able to affect intracellular and extracellular signaling (Perbal 2004). Moreover, these matricellular proteins play central roles in regulating the pathways to the initiation and resolution of normal wound healing and fibrosis in response to multiple forms of injury in many organs (Riser et al. 2015). Furthermore, CCNs as scaffolding proteins can interact with multiple biomolecules and play opposing functions in development, physiology, pathology, and malignancy of many other diseases, making the understanding of their biological functions complex (Kubota et al. 2022).

CCN5 was originally cloned by suppression subtractive hybridization as a gene upregulated in Wnt-1 transformed mouse mammary epithelial cells (i.e., C57MG) that were stably transformed by a Wnt-1 retrovirus (Pennica et al. 1998). It lacks the C-terminal cysteine knot domain found in other CCN family members and is localized mostly in the cytoplasm and in part in the nucleus, allowing to acts as a transcriptional repressor presumably through association with histone deacetylase 1 (Sabbah et al. 2011). Although CCN5 lacks the cysteine knot domain, it was recently demonstrated that CCN5 evolves anti-fibrotic activity in the heart by inducing apoptosis through Smad7 mediated inhibition of the NF-κB signaling pathway especially in myofibroblasts (MFB) but not in cardiomyocytes or fibroblasts (Nguyen et al. 2022). In the respective study, the authors could show that CCN5 tips the balance between p53 and NF-κB over in favor to induce p53-dependent apoptosis by direct induction of Smad7 (Nguyen et al. 2022).

Although *Ccn5* expression was systematically analyzed in rodent embryonic and adult tissues, functional data of CCN5 in the liver is scarce (Jones et al., 2007; Gray et al., 2007). Initially it was shown that CCN5 did not show expression differences in human HCC compared to normal liver samples, neither was there an association of CCN5 expression and clinical features (Zhang et al. 2015). However, in a recent study it was found that CCN5 was downregulated in HCC compared to normal adjacent tissue and this was coupled to poor prognosis. CCN5 was shown to be lower expressed in several HCC cell lines and overexpression reduced migration, invasiveness, proliferation and the expression of epithelial-to-mesenchymal transition (EMT) markers, implying a protective role in vitro (Jia et al. 2021). In contrast, high expression of CCN5 in vivo provokes enhanced infiltration of fibroblasts by triggering the expression of HMGB1 that weakens the anticancer effects (Jia et al. 2021). In addition, CCN5 promotes liver repair by stimulating the in vivo homing of bone mesenchymal stem cells to the injured liver and stimulating endothelial cell proliferation and angiogenesis through modulating Cxcr4 signaling (Qin et al. 2017).

Importantly, it interferes with the transforming growth factor-β (TGF-β) signaling pathway by repressing genes associated with EMT, possibly by restricting expression of the TGF-β type II receptor (Sabbah et al. 2011). In line, a study in which CCN5 was transiently overexpressed in the human hepatic stellate cell line LX-2 showed that elevated expression of CCN5 suppressed Smad2 phosphorylation and expression of α-smooth muscle actin (α-SMA) and collagen type I (Col I), which are hallmarks of liver fibrosis (Zhang et al. 2013). However, there is only limited information available about CCN5 function and expression in different liver cell types.

Here we analyzed the expression of CCN5 in isolated primary liver cells showing that CCN5 is highest expressed in MFB derived from hepatic stellate cells or portal fibroblasts. In line, CCN5 expression is increased in the cholestatic liver injury model induced by bile duct ligation. Adenoviral overexpression of CCN5 in portal myofibroblasts results in reduced Col I expression accompanied by reduced expression of the TGF-β-receptor I and lower activation of Smad2. Although we couldn’t show a direct impact on TGF-β1 signaling upon CCN5 overexpression in pMF, forced CCN5 expression leads to transient unfolded protein response (UPR) induction before cells are finally committed to apoptosis.

## Materials and methods

### Animals

The animals used for isolation of primary liver cells were kept in accordance with the recommendations of the Federation of European Laboratory Animal Science Associations (FELASA). Wild type Sprague–Dawley rats were housed in a 12:12 light–dark cycle at constant humidity (50%) and temperature (20 °C) with free access to food and water ad libitum. Bile duct ligation in rats (n = 5) was performed following protocols that were described previously using Sham-operated animals as control (n = 4) (Arias et al. 2003). All experiments were approved under permit number 81-02.04.2020.A228 by the Review Board for the Care of Animal Subjects of the district government (LANUV, Recklinghausen, North Rhine-Westphalia, Germany).

### Isolation and culturing of primary liver cells

Portal myofibroblasts (pMF) were isolated and cultured following protocols described elsewhere (Borkham-Kamphorst et al. 2018). Primary hepatic stellate cells (HSC) were isolated from male Sprague–Dawley rats through density gradient centrifugation technique on a Nycodenz gradient and cultured as described (Schäfer et al. 1987; Fehrenbach et al. 2001). MFBs were obtained by sub-cultivation of HSC on day 7 of initial culturing. pMF, HSC and MFB were cultured in Dulbecco’s modified Eagle’s medium (DMEM) supplemented with 10% fetal bovine serum (FBS), 4 mM L-Glutamine, 1 mM sodium pyruvate, and 1 × Penicillin/Streptomycin. Primary hepatocytes were isolated according to the collagenase method of Seglen (Seglen 1976) and seeded in collagen-coated petri dishes using serum-free HepatoZYME-SFM medium (Gibco Life Technologies, ThermoFisher Scientific, Merck, Schwerte, Germany). If not otherwise indicated, the experiments depicted are representatives of experiments that were done three times.

### RNA extraction and RT-qPCR

Total cellular RNA from cultured cells was isolated and cleaned with PureLink RNA Mini kits (Invitrogen, Thermo Fisher Scientific) according to manufacturer’s guidelines including an on column DNAse digestion. RNA extraction of liver specimens was performed as described previously through a standard phenol–chloroform extraction, isopropanol precipitation and DNAse digestion (Borkham-Kamphorst et al. 2020). Thereafter, the RNA was cleaned with PureLink RNA Mini kits (Invitrogen, Thermo Fisher Scientific) according to manufacturer’s guidelines (Borkham-Kamphorst et al. 2020). Primers for quantitative real-time PCR (RT-qPCR) were selected from the sequences deposited in the GenBank database using the online ProbeFinder Software (Universal Probe Library Assay Design Center, Roche, Mannheim, Germany). First-strand cDNA was synthesized from 1 µg RNA in 20 µl volume using SuperScript™ II reverse transcriptase and random hexamer primers (Invitrogen). For RT-qPCR, cDNA derived from 50 ng RNA (5 μl of 1:5 dilution of cDNA) was amplified in 25 µl volume using SYBR^®^ GreenER™ qPCR SuperMix for ABI PRISM^®^ (Invitrogen) in a TaqMan PCR machine. The PCR conditions were set to 50 °C for 2 min, 95 °C for 10 min initial denaturation, followed by 40 cycles of 95 °C for 15 s, and 60 °C for 1 min. Relative mRNA expression was normalized to the housekeeping gene glyceraldehyde 3-phosphate dehydrogenase (*GAPDH*) and calculated using the 2^−ΔΔCT^ method (Schmittgen and Livak 2008). All primers used in this study are listed in Suppl. Table 1.

### Protein extraction and Western blot analysis

For protein extraction, cells were washed in PBS and lysed in RIPA buffer (50 mM Tris–HCl (pH 7.2), 150 mM NaCl, 1% (w/v) NP-40, 0.1% (w/v) SDS, 0.5% (w/v) sodium deoxycholate) containing cOmplete™-proteinase inhibitor (#11697498001, Merck KGa, Darmstadt, Germany) and phosphatase inhibitor cocktail II (#P5726, Sigma-Aldrich, Taufkirchen, Germany). The protein concentration in each sample was quantified using DC™ Protein assay kit I (Bio-Rad, Feldkirchen, Germany) according to the manufacturer’s instructions. Equal amounts of proteins were diluted with Nu-PAGE™ LDS electrophoresis sample buffer (Invitrogen) containing 50 mM dithiothreitol (DTT) as a reducing agent. The samples were heated at 80 °C for 10 min and separated in 4–12% Bis–Tris gradient gels, using MES running buffer (all reagents were obtained from Invitrogen). Proteins were electroblotted onto nitrocellulose membranes (Schleicher & Schuell BioScience GmbH, Dassel, Germany) and transfer confirmed by Ponceau S staining. Non-specific binding sites were blocked with 5% (w/v) non-fat milk powder in Tris-buffered saline and 0.1% Tween 20 (TBST). All antibodies (Suppl. Table 2) were diluted in 2.5% (w/v) non-fat milk powder in TBST. Primary antibodies were detected using horseradish peroxidase (HRP)-conjugated anti-mouse-, anti-rat-, anti-rabbit-, or anti-goat IgG (all from Invitrogen) and the SuperSignal chemiluminescent substrate (Pierce, Bonn, Germany). The relative quantity of protein bands from Western blots was determined by measuring the density of each signal using the ImageJ image processing program developed from the National Institute of Health and the Laboratory for Optical and Computational Instrumentation (Schneider et al. 2012).

### Testing for antibody specificity

For transient transfection with plasmids encoding mouse or human CCN5 proteins, human HEK293 cells (#CRL-1573, ATCC, Manassas, VA, USA) were plated into 6-well plates and grown to about 60–70% confluence. The medium was renewed and cells transfected with 2 µg purified plasmid DNA using 6 µl FuGENE^®^6 (Roche) as transfection reagent. 16 h later, the medium was renewed and protein extracts prepared after an additional 24 h culture period. Subsequent Western blot analysis was done as described above. Equal protein loading was demonstrated by Ponceau S stain and probing membranes with an antibody directed against β-actin. The vectors used for transfection were Myc-DDK-tagged pCMV6-Entry-mCCN5 (#MR203197, Origene, Herford, Germany) and Myc-DDK-tagged pCMV6-Entry hCCN5 (#RC204636, Origene). A FLAG-tagged control vector pcDNA-CAR-FLAG was used as a control.

### Cloning strategy of CCN5 adenoviral constructs and adenoviral infection

The two expression vectors RC204636 and MR203197 encoding full-length human or mouse Myc-DDK-tagged CCN5/WISP2 were obtained from Origene. For cloning adenoviral expression vectors the respective vectors were digested with *Bam*HI/*Not*I and cloned into the adenoviral shuttle vector pShuttle-CMV, which was cut with *Bgl*II/*Not*I and dephosphorylated with alkaline phosphatase. The integrity of cloning sites was determined by standard sequencing. Thereafter, the fragments containing the entire coding sequences of CCN5 were transferred into the adenoviral backbone vector following standard procedures. Adenoviral vectors were amplified and purified as described elsewhere (Meurer et al. 2019). All plasmids used for cloning the final adenoviral CCN5 expression constructs are depicted in Suppl. Figure 1.

## Results

### Testing for CCN5 antibody specificity

CCN5 belongs to a family of highly homologous secreted proteins. To evaluate the specificity of five different commercially available CCN5 antibodies, we first performed Western blot analysis. For overexpression, HEK293 cells were transfected with vectors encoding c-myc- and FLAG-tagged murine or human CCN5 and the corresponding cell extracts and supernatants were analyzed by Western blot (Suppl. Figure 2). The antibody from Abcam (ab38317), which is specified for human CCN5 detected human and mouse CCN5, which however was not effectively secreted from the transfected HEK293 cells. The Sigma antibody (SAB1401444) directed against human CCN5 showed only low reactivity and had a general high background staining. The LSbio antibody (LS-C349158) announced as an antibody that should detect human, mouse and rat CCN5 identified human CCN5 and mouse CCN5 with lower signals. However, this antibody also showed high background staining. The Bioss antibody (bs5100R) was inappropriate, while the Santa Cruz antibody (sc-12010) detected murine CCN5 with high affinity without cross-reactivity to human CCN5. To confirm the expression of the recombinant CCN5 proteins, we further probed the Western blots with antibodies specific for the c-myc and the FLAG tag. Based on this analysis, we decided to work only with the CCN5 antibody from Abcam that gave the best signals with the lowest background.

### Endogenous expression of CCN5 in cultured liver cells

**Fig. 1:**
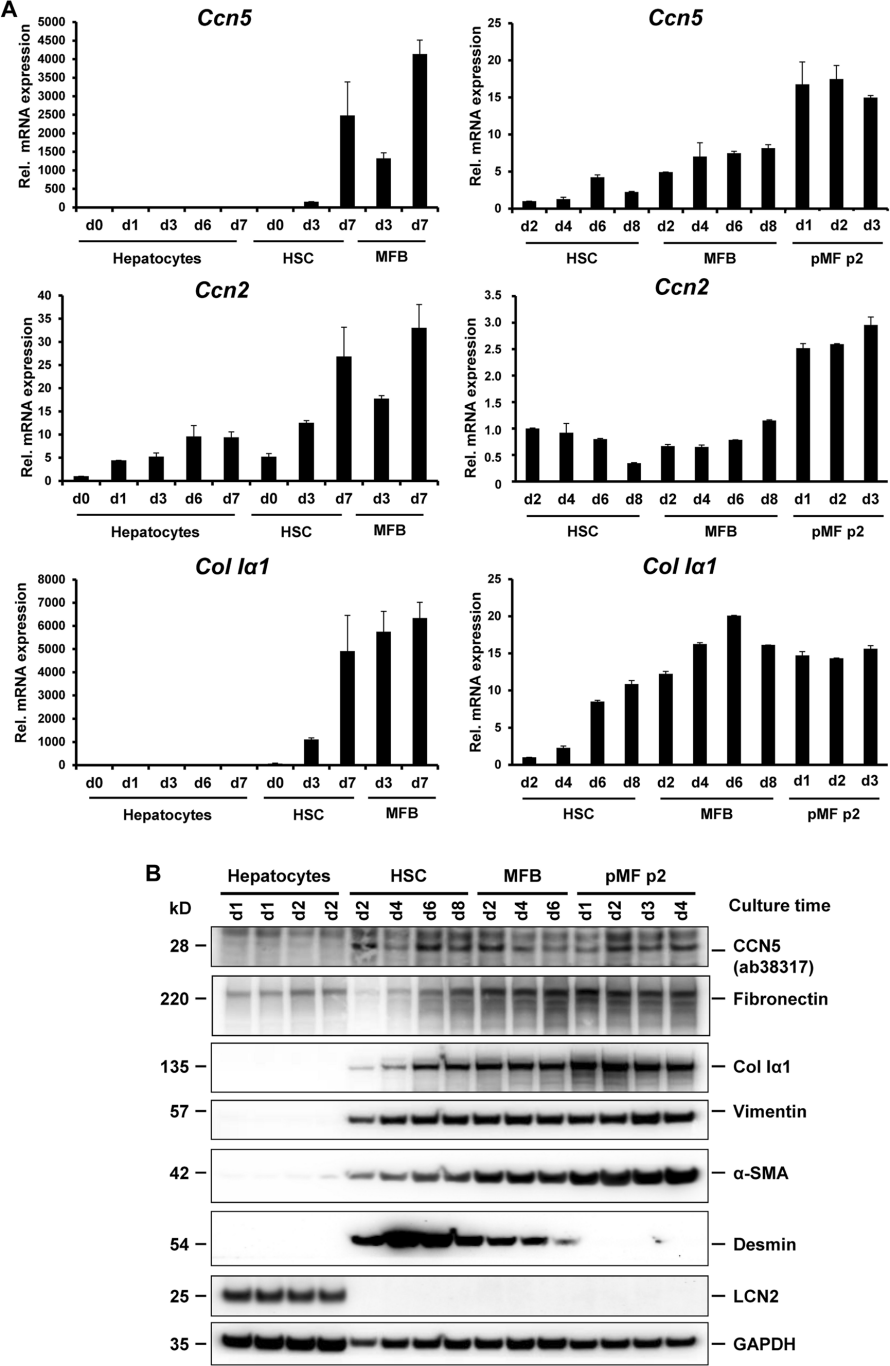
Expression kinetics of *Ccn5*, *Ccn2*, and *Col Iα1* in isolated liver cells. (**A**) mRNA expression of *Ccn5, Ccn2*, and *Col Iα1* in hepatocytes, hepatic stellate cells (HSC), myofibroblasts (MFB) and portal myofibroblasts (pMF) in passage 2 (p2) cultured  for the indicated time intervals was determined by RT-qPCR. *Ccn5* and *Col Iα1* expression is undetectable in hepatocytes, while in contrast *Ccn2* mRNA increases during prolonged culture times in hepatocytes. In activated HSC and transdifferentiated MFB *Ccn5* mRNA is up-regulated with the activation state of the cells. This up-regulation is even more pronounced than the expression of *Ccn2* and parallels the induction of *Col Iα1* in the course of HSC to MFB transition. Activated pMF displaying a MFB-like phenotype express large quantities of *Ccn5*, *Ccn2*, and *Col Iα1* mRNAs. (**B**) Protein analysis confirms the absence of CCN5 in hepatocytes and the expression in HSC/MFB and PMF. In this analysis, Vimentin and α-SMA served as markers to demonstrate the mesenchymal phenotype of HSC, MFB and pMF. LCN2 was used as a marker for hepatocytes. Fibronectin and *Col Iα1* expression confirmed elevated expression of extracellular matrix proteins during transition of HSC to MFB, while the discrimination between pMF and HSC/MFB was done by probing for Desmin

To assess the basal expression of *Ccn5* in primary liver cells, we isolated total RNA of rat hepatocytes, HSC, MFB, and pMF cultured for different times and tested for *Ccn5* expression by RT-qPCR (Fig. 1A). This analysis revealed that *Ccn5* mRNA is similarly expressed to collagen type I α1 (*Col Iα1*) in HSC, MFB and pMF, while the expression was virtually absent in primary hepatocytes, suggesting that *Ccn5* is a marker of mesenchymal profibrogenic cells. Therefore, *Ccn5* shows a similar expression pattern to *Ccn1*, *Ccn3*, and *Ccn4*, which are also primarily expressed in mesenchymal/profibrogenic cells (Suppl Fig. 3). On the other hand, *Ccn2* is not only expressed in mesenchymal cells, but also in hepatocytes.

The expression of CCN5 in HSC, MFB and pMF was also confirmed by Western blot analysis, in which CCN5 showed the same cellular specificity for profibrogenic cells such as collagen type I, Vimentin, α-smooth muscle actin (α-SMA) (Fig. 1B). To demonstrate the purity of isolated cell subpopulations in this set of experiments, we further tested for expression of Lipocalin 2 (LCN2), which in liver is predominantly expressed in hepatocytes and desmin, which represents a marker for HSC/MFB but is not expressed in hepatocytes and pMF. To further characterize the authenticity and purity of the isolated cells, we performed densitometrical analysis of marker protein expression discriminating epithelial (hepatocytes) and mesenchymal cells (HSC/MFB, pMF) (Suppl. Figure 4) as well as RT-qPCR for markers of HSC/MFB- and pMF-derived cells (Suppl. Figure 5). HSC, MFB and pMF were confirmed to express *Acta2* and Vimentin. In addition, HSC and MFB expressed GFAP and desmin that are typical markers for these cells (Wang et al. 2009; Acharya et al. 2021). Moreover, pMF were positive for fibronectin 1, while elastin and mesothelin was detected in all three cell types. The thymus cell antigen-1 (*Thy-1*), representing a marker for pMF (Katsumata et al. 2017), was predominantly expressed in pMF. In addition, (cyto)keratin 19 (*Krt19*) indicating the mesenchymal stem cell feature of pMF (Dudas et al. 2007; Yovchev et al. 2009; Lei et al. 2022) and Il-6 that is also a useful marker to identify pMF in the myofibroblast population (Karin et al. 2016) was majorly found in pMF.

### Expression of CCN5 in fibrotic liver is induced by cholestasis

**Fig. 2:**
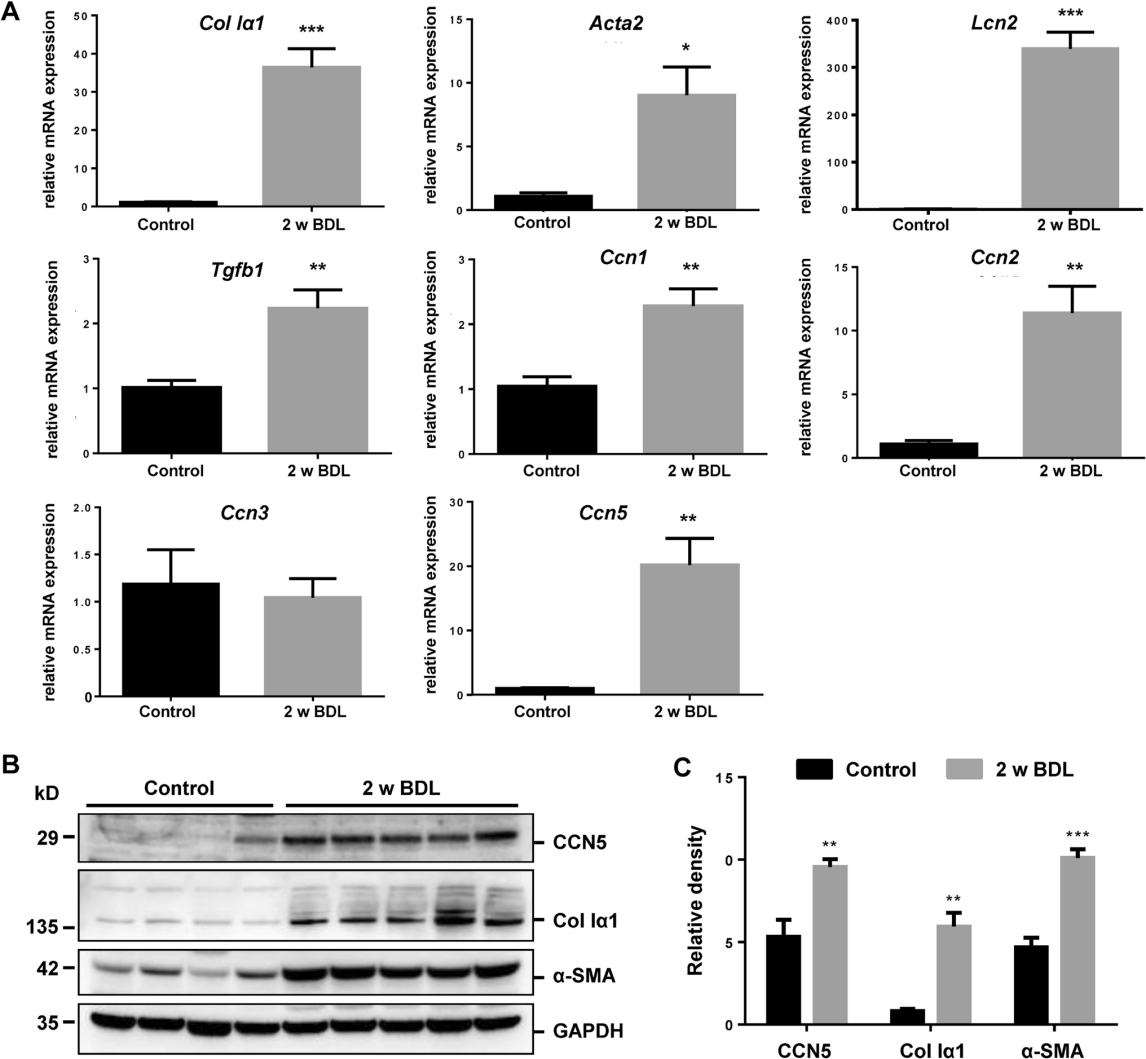
*Ccn5* expression in the inflammatory/fibrogenic bile duct obstruction model. (**A**) Mice were subjected to bile duct ligation (BDL) for 2 weeks. Hepatic expression of indicated genes was compared to sham-operated control animals by RT-qPCR showing that *Col Iα1*, *Acta2*, *Lcn2*, and *Tgfb1* are strongly upregulated during hepatic injury. Similarly, the expression of *Ccn1* and *Ccn2* in fibrotic liver tissue is increased, while the expression of *Ccn3* is comparable in healthy and diseased livers. Interestingly, the expression of *Ccn5* is significantly upregulated in diseased livers. Differences between the groups reaching significance are marked by asterisks (*p < 0.05, **p < 0.01, ***p < 0.001). (**B**) Protein analysis confirms the increased expression of α-SMA and Col Iα1 and the elevated expression of CCN5 in livers of animals subjected to BDL. (**C**) Densitometric analysis of Western blot data depicted in (**B**)

The expression analysis demonstrated that CCN5 mRNA and protein is highly expressed in pMF (cf. Figure 1). Since pMF play a major role in the setting of cholestatic liver diseases, we next analyzed *Ccn5* expression in rat fibrotic livers that were obtained by subjecting animals to bile duct ligation (BDL) for two weeks. This analysis revealed that *Ccn5* expression was significantly upregulated (*p* = 0.0051) during liver fibrogenesis similar to *Col Iα1* (*p* = 0.0011), α-SMA (*Acta2*, *p* = 0.0002) and *Tgfb1* (Fig. 2A). Elevated expression was also found for *Ccn1* and *Ccn2*, which confirms previous findings (Kim et al. 2013; Borkham-Kamphorst et al. 2016a; Gressner et al. 2007). In contrast, the expression of *Ccn3* was not altered. The inflammatory response in the liver subjected to BDL was also indicated by a significant increase of Lipocalin 2 (*Lcn2*) expression, a finding that we have reported before (Borkham-Kamphorst et al. 2011). Increased expression of CCN5, collagen type I, and α-SMA during hepatic fibrosis was also demonstrated by Western blot analysis (Fig. 2B,C).

### Biological effects of CCN5 overexpression in portal myofibroblasts

**Fig. 3:**
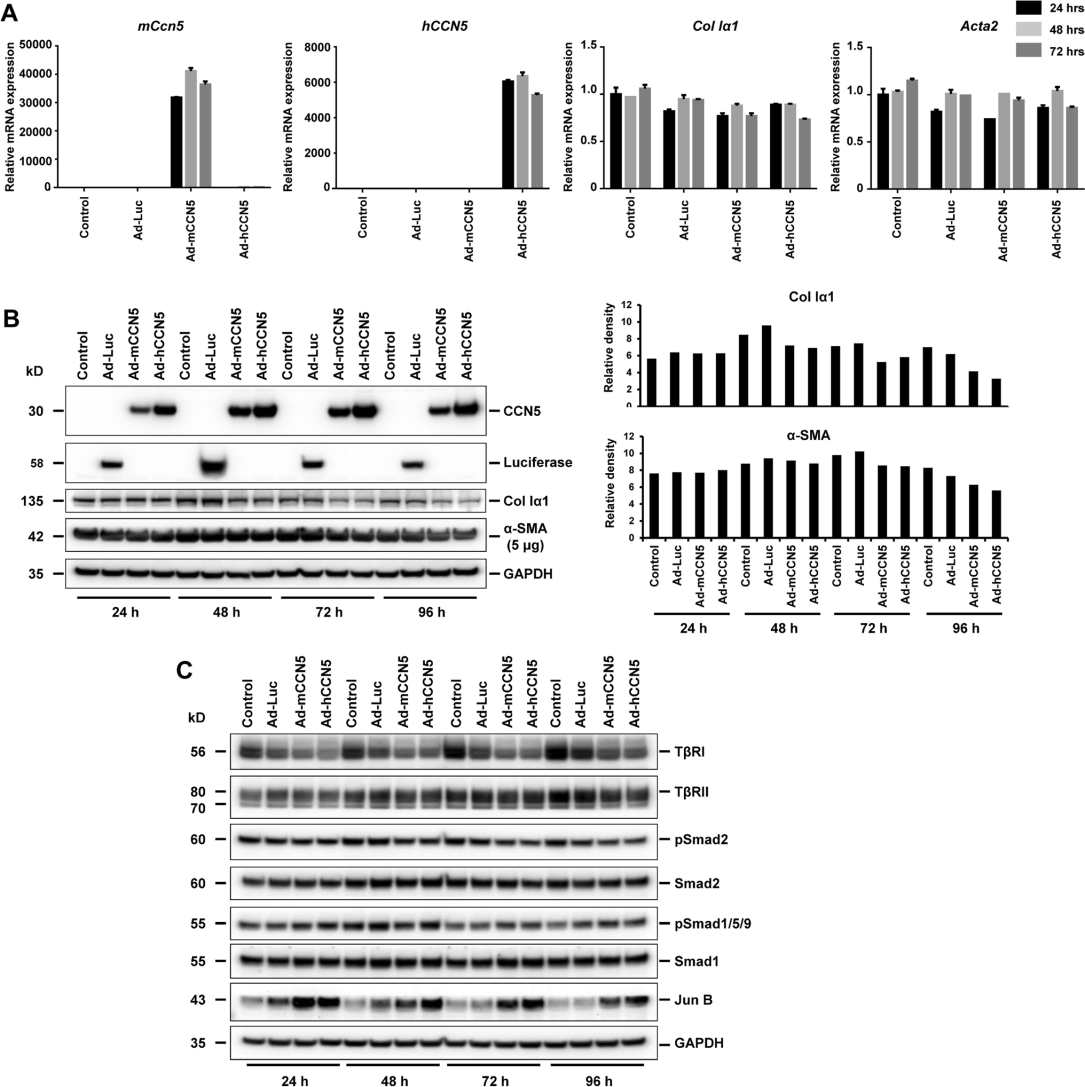
Effects of adenoviral overexpression of CCN5 in portal myofibroblasts. (**A**) Cultured portal myofibroblasts were infected for indicated time intervals with adenoviral expression vectors directing expression of murine CCN5, human CCN5 or Luciferase as a control. Mock infected cells served as a further control. Total RNA was isolated and the expression of *mCcn5*, *hccn5*, *Col Iα1*, and *Acta2* determined by RT-qPCR. (**B**) Protein analysis confirms the prominent overexpression of the transgenes (CCN5, Luciferase). Both CCN5 transgenes cause a reduction in *Col Iα1* expression after infection for 48 h, 72 h, and 96 h, as evaluated by densitometric analysis of the depicted Western blot results. On the other hand, the effect on α-SMA expression is much less, leading to a lower expression only at 72 h and 96 h. (**C**) Western blot analysis demonstrates that overexpression of CCN5 results in reduced expression of the TGF-β type I receptor (ALK5, TβRI) and TGF-β type II receptor (TβRII). As a mutual consequence of the lowered receptor expression, Smad2 gets less activated from 48 h on, while at 72 h and 96 h after overexpression of CCN5 the genuine BMP or „alternative “ TGF-β1/Smad pathway as assessed by increased Smad1/5/8 phosphorylation is activated. In addition, Jun B is strongly up-regulated in cells overexpressing CCN5. GAPDH expression was used in Western blot analysis to document equal protein loading. Depicted is a representative experiment of an analysis that was done twice. The densitometric analysis of the Western blot is shown in Suppl. Figure 6

**Fig. 4:**
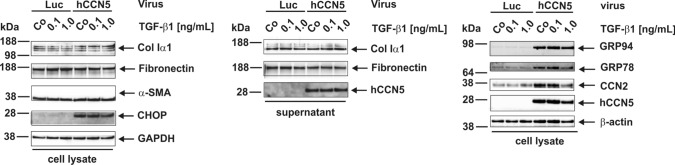
Impact of CCN5 on TGF-β signaling. Portal myofibroblasts were infected with adenoviral vectors expressing either human CCN5 (hCCN5) or the luciferase reporter gene (Luc). After 48 h, the cells were stimulated with the indicated concentrations of recombinant TGF-β1. Protein extracts and supernatants were prepared and tested for expression of Col Iα1, Fibronectin, α-SMA, CHOP, GAPDH (*left panel, cell lysate*), Col Iα1, Fibronectin, hCCN5 (*middle panel, culture supernatant*), and GRP94, GRP78, CCN2, and CCN5 (*right panel, cell lysate*). The expression of β-actin was used to document equal protein loading in each lane. Of note, there was no significant effect on the extracellular matrix components Col Iα1, Fibronectin and the activation marker α-SMA, but a strong TGF-β1 independent up-regulation of the UPR related proteins GRP94, GRP78 and CHOP. The densitometric analysis of the three Western blots is shown in Suppl. Figure 7

For analysis of CCN5 functions, we decided to overexpress the respective protein in pMF. To effectively force expression of the CCN5 protein in cultured primary pMF, we cloned adenoviral expression vectors that constitutively express human or mouse CCN5 proteins under control of a CMV promoter (cf. Suppl. Figure 1). Both constructs were able to direct high expression of CCN5 but had no direct effect on collagen type I or *Acta2* mRNA expression that are important for myofibroblast function. However, there was a slight trend to reduce cellular collagen I quantities (Fig. 3A,B). Nevertheless, the expression of transforming growth factor-β type I (TβRI), that is the key regulator in initiating fibrogenic responses by activating intracellular Smad mediators and triggering fibrogenic marker protein expression, was reduced and JunB expression was increased in cells overexpressing CCN5 (Fig. 3C, Suppl. Figure 6). When cells were further stimulated with TGF-β1, the overexpression had no significant impact on the ECM components. The expression and secretion of Col Iα1 and Fibronectin was not changed in the individual experiments/replicates in stimulatory conditions (Fig. 4, *left and middle panels*). Nevertheless, α-SMA expression was slightly increased in the presence of hCCN5 (Fig. 4, *left panel*). On the other hand, hCCN5 overexpression resulted in increased quantities of the 94-kD glucose-regulated protein (GRP94), and GRP78 compared to cells that were infected with the luciferase reporter gene vector (Fig. 4, *right panel*) in a ligand dose-dependent manner. In addition, the expression of the damage-inducible transcript 3, also known as C/EBP homologous protein (CHOP) acting as a pro-apoptotic transcription factor was increased by CCN5 virtually independent of TGF-β1 as best documented in the densitometric analysis of respective Western blot results (Suppl. Figure 7).

### CCN5 expression provokes endoplasmic reticulum stress and unfolded protein response

**Fig. 5:**
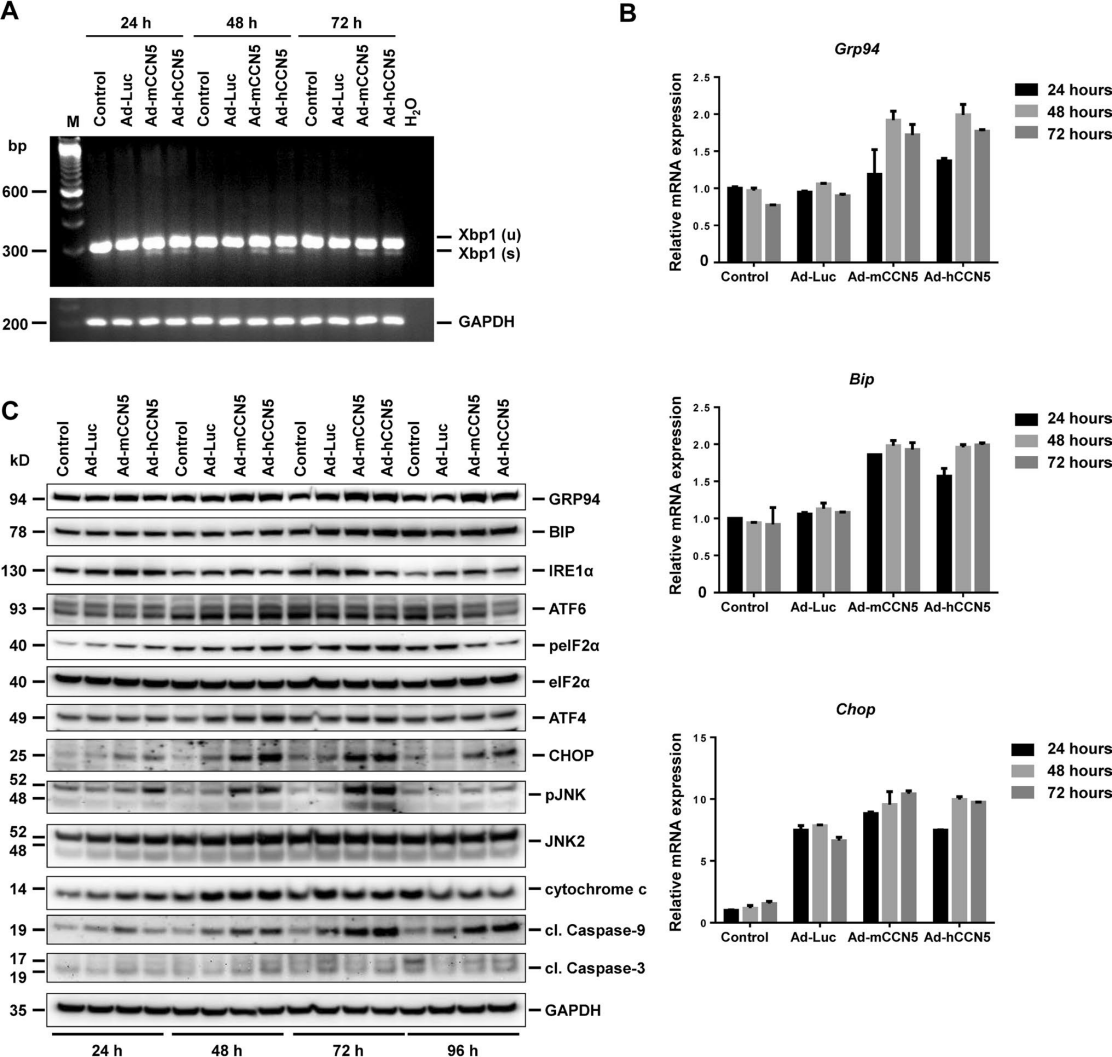
CCN5 induces endoplasmic reticulum stress in portal myofibroblasts. (**A**) Portal myofibroblasts were infected with indicated adenoviral constructs for indicated time intervals. Subsequently, total RNA was isolated, cDNA synthesized. The expression of unspliced (u) and spliced (s) *Xbp1* expression after infection with adenoviral vectors expressing murine or human CCN5 was analyzed by semi-quantitative PCR. Spliced *Xbp1* (Xbp1 (s)) transcripts were found in the presence of overexpressed mCCN5 and hCCN5, while cells that were infected with a control virus (Ad-Luc) or cells that were left uninfected showed no spliced *Xbp1*. In this analysis, GAPDH was taken as a loading control. (**B**) Relative mRNA expression of *Grp94*, *Bip* and *Chop* as assessed by RT-qPCR in samples taken from (**A**). (**C**) Western blot analysis of GRP94, GRP78/BIP, IRE1α, ATF6, peIF2α, eIF2α, ATF4, CHOP, pJNK, JNK2, cytochrome c, cleaved caspase-9, and cleaved caspase-3 in protein samples taken from portal myofibroblasts that were infected with indicated adenoviral vectors for indicated time intervals. Expression of GAPDH was used to demonstrate equal protein loading in each lane. The densitometric analysis of the Western blot is given in Suppl. Figure 8

We have previously shown that CCN1 has the capacity to induce cellular senescence and apoptosis in primary pMF and HSC (Borkham-Kamphorst et al. 2014; Borkham-Kamphorst et al. 2016a). Moreover, we have shown that the transient overexpression of CCN1, CCN2, CCN3 and CCN4 induced ER stress and UPR in pMF (Borkham-Kamphorst et al. 2016b). To evaluate if CCN5 has the same biological activity in inducing ER stress, we next infected pMF with the adenoviral CCN5 expression vectors using an adenoviral reporter vector expressing Luciferase as a control. Importantly, both CCN5 proteins induced the unconventional splicing of *Xbp1* mRNA, which in turn can be translated into the potent transcription factor XBP1s that promotes the transcription of UPR-related genes encoding ER chaperones and folding enzymes (Uemura et al. 2009) (Fig. 5A). The production of *Xbp1 (s)* mRNA was associated with a slight (but not significant) increase of glucose-regulated protein 94 (*Grp94*) and GRP78 (*Bip*) mRNAs (Fig. 5B) and higher GRP78 protein expression, that together are hallmarks of the UPR response (Marzec et al. 2012). In addition, the adenoviral introduced transgenes enhanced the expression of C/EBP homologous protein (CHOP) mRNA and protein, representing a multifunction transcription factor associated with formation of ER stress. Moreover, cells overexpressing CCN5 had elevated levels of cleaved caspase-9 protein expression acting as a critical initiator of intrinsic apoptosis, physiological cell death and pathological tissue degeneration (Avrutsky and Troy 2021). Finally, the cells showed increased phosphorylation of JNK (Fig. 5C, Suppl. Figure 8), that in its activated form promotes intrinsic apoptosis by multiple mechanisms (Dhanasekaran and Reddy 2017).

## Discussion

There is a large body of evidence showing that members of the CCN protein family have crucial functions in many areas, such as control of development, cell fate, angiogenesis, cell adhesion, migration, mitogenesis, cell survival, and tumorigenesis. In addition, numerous independent studies have shown that different CCN proteins are crucially impacting the pathogenesis of fibrosis (Weiskirchen 2011; Chen and Brigstock 2017). However, the biological functions of CCN proteins in the pathogenesis of fibrotic liver lesions are complex and partially opposite.

In particular, CCN2 is increasingly expressed in activated HSC, which directly promotes the expression of extracellular matrix proteins. A recent study has shown that conditional knockout in mice and hepatocyte-specific deletion of CCN2 in rats resulted in reduced expression of fibrosis-related genes including Slit, α-SMA, and collagen type I when subjected to peri-central hepatocyte damage caused by carbon tetrachloride intoxication (Pi et al. 2022). On the contrary, CCN1 is upregulated during hepatic injury acting as an inhibitor of liver fibrosis by triggering induction of reactive oxygen species, cellular senescence, apoptosis and reduced TGF-β signaling in activated HSC and portal fibroblasts (Kim et al. 2013; Borkham-Kamphorst et al. 2014). However, another report has demonstrated that CCN1 expressed by HSC is involved in the progression from cirrhosis to hepatocellular carcinoma (HCC) through promoting the growth and proliferation of HCC (Li et al. 2018).

In hepatic fibrosis, CCN3 is increased expressed and mainly found in non-parenchymal cells, stimulate the migration of HSC and induce hepatocyte apoptosis, while the suppression of CCN3 enhanced expression of profibrotic marker proteins in primary HSC (Borkham-Kamphorst et al. 2012a, 2012b). Moreover, increased CCN3 is associated with metastasis, more severe cirrhosis, increased EMT, and further inversely related to the prognosis of HCC (Li et al. 2019).

In the liver, *ccn4*  mRNA expression is positively correlated with body mass index and hepatic expression of fibrotic and inflammatory marker genes and recombinant CCN4 protein caused dose-dependent induction of matrix metalloproteinase 9 (MMP9) involved in the breakdown of extracellular matrix and stimulated the expression of MCP1 representing a potent chemoattractant during inflammatory hepatic disease (Pivovarova-Ramich et al. 2021). Moreover, it further potentiated TGF-β-mediated expression of profibrogenic markers in an immortalized HSC cell line (Jian et al. 2014; Pivovarova-Ramich et al. 2021). In line, the blockade of CCN4 expression using a monoclonal anti-CCN4 antibody attenuated experimental liver fibrogenesis (Li et al. 2015).

On the contrary, CCN6 expression is downregulated in livers of experimental fatty liver disease and patients suffering from non-alcoholic steatohepatitis (NASH), while the overexpression of CCN6 significantly attenuated hepatic steatosis, inflammation, and fibrosis in NASH mice suggesting that this CCN member has antifibrogenic activities (Song et al. 2022).

Actually, there is very little known about CCN5 biology in hepatic disease and the few previous articles available are somewhat inconsistent. CCN5 expression is lowered in HCC tumor tissues compared with normal tissue, while contrarily high CCN5 expression was associated with better prognosis in HCC, suggesting a dual role in CCN5 activity (Jia et al. 2021). Possibly, these beneficial effects might be related to the fact that CCN5 promotes the homing of bone marrow-derived mesenchymal stem cells, which promote liver regeneration and repair (Qin et al. 2017). However, the precise biological function of CCN5 in the different liver cell types, liver homeostasis, and hepatic injury are still unknown.

In the present study, we first determined the expression of CCN5 in different primary rat liver cells, showing that CCN5 is highest expressed at the mRNA and protein level in pMF and HSC/MFB and nearly absent in hepatocytes (cf. Figure 1A, Suppl. Figure 4). This is also reflected at the protein level, in which expression of CCN5 is virtually absent in hepatocytes (Fig. 1B). The purity and identity of cultured primary cells was documented by expression of specific marker genes including collagen type I, Vimentin and α-SMA that are specific for HSC, MFB and pMF. Furthermore, the expression of desmin was taken to discriminate between the HSC/MFB fraction and pMF, while the expression of LCN2 was used as a marker of hepatocytes and expression of Thy-1, Krt19 and Il-6 was taken as a characteristic of pMF (Suppl. Figure 5).

Next we analyzed CCN5 expression in fibrotic livers that were obtained by subjecting mice to BDL for two weeks. This analysis revealed that CCN5 was markedly upregulated during hepatic fibrosis and comparable to collagen type I, α-SMA and TGF-β1 that are well-established markers which are increasingly expressed during hepatic fibrosis (Fig. 2) (Acharya et al. 2021). In addition, we confirmed elevated expression of *Ccn1* and *Ccn2* in damaged and fibrotic livers, a finding that was reported by us and others before (Kim et al. 2013; Borkham-Kamphorst et al. 2016a; Gressner et al. 2007).

To clarify if CCN5 has direct effects on the expression of profibrogenic markers such as collagen type I and α-SMA, we next overexpressed CCN5 in pMF. Although the overexpression had no or marginal influence on the mRNA expression of respective profibrogenic markers in pMF (Fig. 3A), we found that the transgenes effectively reduced collagen type I protein expression (Fig. 3B). In line with a reduced collagen I expression, the TβRI expression was down-regulated and the downstream mediator Smad2 less phosphorylated (Fig. 3C). The serine/threonine protein kinase TβRI is most critical in transducing TGF-β signals from the cell surface to the cytoplasma, potentially indicating that CCN5 might interfere with general TGF-β signaling in pMF. In addition, JunB was strongly up-regulated. The protooncogene JunB is an AP-1 component and profibrogenic transcription factor involved in acute liver inflammation and increased in activated HSC and positively correlated with liver fibrosis (Smart et al. 2006). This suggests that the increase of CCN5 expression during hepatic fibrosis might be an intrinsic and complex mechanism that can either block or promote aspects of fibrogenesis in pMF. In addition, the overexpression of CCN5 resulted in basal higher but reduced induction of CCN2 by TGF-β1 (Fig. 4), which represents a CCN protein that evolves profound fibrotic activities (Gressner et al. 2009; Weiskirchen 2011; Ramazani et al. 2018; Pi et al. 2022).

Most strikingly, elevated quantities of CCN5 provoke ER stress and UPR in pMF indicated by the conversion of unspliced *Xbp1* (Xbp1 (u)) to the spliced form (Xbp1 (s)) (Fig. 5A), increased expression of *Grp94* and *Grp78*/*Bip* mRNAs (Fig. 5B), and elevated expression of GRP94, CHOP, and cleaved caspase-9 proteins (Figs. 4, 5C). The capacity of CCN5 to induce ER stress and UPR is shared with other CCN members. We have previously shown that the overexpression of CCN1, CCN2, CCN3 and CCN4 provokes ER stress and UPR (Borkham-Kamphorst et al. 2018). Since all CCN proteins exhibit the same modular organization, except that CCN5 is missing the cysteine knot CT-domain, and shares a highly related amino acid sequence in which 38 cysteine residues are conserved, it might be possible that an excess of a CCN protein that requires proper disulfide-bond formation and protein folding prior to secretion might overstress the capacity of the cellular synthesis protein apparatus and provoke ER stress and UPR. As a consequence, pMF might undergo apoptosis, preventing the excess formation of extracellular matrix components. As such, CCN proteins are potentially the missing link in driving apoptosis and the disappearance of pMF in rat and human cultured liver slices derived from fibrotic livers during fibrotic liver remodeling (Guyot et al. 2007).

ER stress and UPR have nowadays recognized as a major contributor to liver disease and hepatic fibrosis (Maiers and Malhi 2019). A better understanding of the biological activities of CCN proteins and the involvement of CCN proteins in inducing ER stress and UPR might provide potential new therapeutic modalities by which controlled apoptosis might be triggered in overactive fibrogenic cells to halt overshooting fibrotic responses.

In sum, hepatic CCN5 expression is majorly found in profibrogenic cells (i.e., HSC, MFB, pMF) and the expression of this matricellular protein is significantly increased during liver fibrogenesis similar to that of *Col Iα1*, *Acta2*, *Tgfb1*, *Ccn1*, and *Ccn2*. The finding that the overexpression of CCN5 reduced Col Iα1 protein expression and further reduced expression of TβRI and downstream TGF-β signaling might indicate that CCN5 is a key factor counteracting overshooting fibrosis within the liver. In addition, the finding that CCN5 has the capacity to induce endoplasmic reticulum stress and UPR in portal fibroblasts might shed light on the mechanisms by which CCN5 ameliorate fibrosis on the cellular level. Although we have not tested yet if elevated CCN5 expression during hepatic fibrosis correlates with increased CCN5 serum levels, respective measurements might be diagnostically relevant to estimate the activity, progress or outcome during ongoing hepatic fibrosis. In this regard CCN5 might complement the list of cellular communication network proteins that are useful biomarkers in translational research.

## Supplementary Information

Below is the link to the electronic supplementary material.

Supplementary file1 (DOCX 2246 KB)

## Abbreviations

α-SMA: α-Smooth muscle actin
BDL: Bile duct ligation
CCN: Cellular communication network
Col I: Collagen type I
ECM: Extracellular matrix
EMT: Epithelial-to-mesenchymal transition
HCC: Hepatocellular carcinoma
HSC: Hepatic stellate cell(s)
MFB: Myofibroblast(s)
pMF: Portal myofibroblast(s)
TBST: Tris-buffered saline and 0.1% Tween 20
TGF-β: Transforming growth factor-β
UPR: Unfolded protein response
