# Supplementary material for: Expression and biological function of the cellular communication network factor 5 (CCN5) in primary liver cells
Source: J Cell Commun Signal. 2023 May 11;17(2):307–20. doi: 10.1007/s12079-023-00757-8 (PMC10326238; doi:10.1007/s12079-023-00757-8)
Supplement: Supplementary file 1 — Supplementary file1 (DOCX 2246 KB) [file 12079_2023_757_MOESM1_ESM.docx]

# Supplements

#
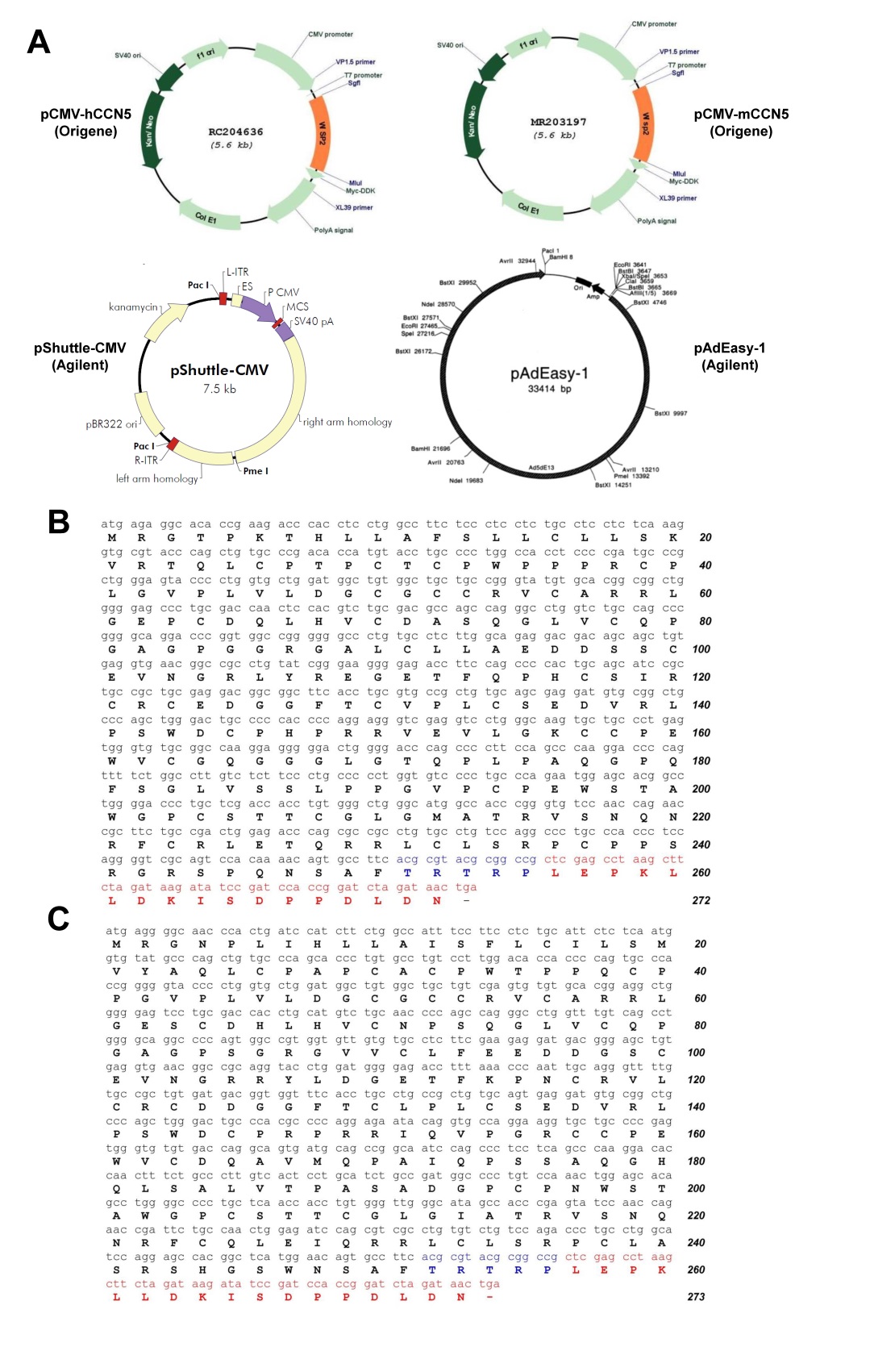


# Suppl. Figure 1: Vectors used for cloning of adenoviral expression vectors for mouse and human CCN5. The expression vectors RC204636 and MR203197 for human and mouse CCN5 were obtained from Origene. The shuttle vector pShuttle-CMV and the adenoviral backbone vector pAdEasy-1 were obtained from Agilent Technologies. The final adenoviral expression vectors encode for full-length CCN5 proteins that carry an artificial 22-amino acid stretch at their C-termini that originates from the original expression vectors (*blue*) or the shuttle vector (*red*). For details about the cloning of adenoviruses AdEasy-1-CMV-hCCN5 and pAdEasy-1-CMV-mCCN5 see Material and Method section.

#
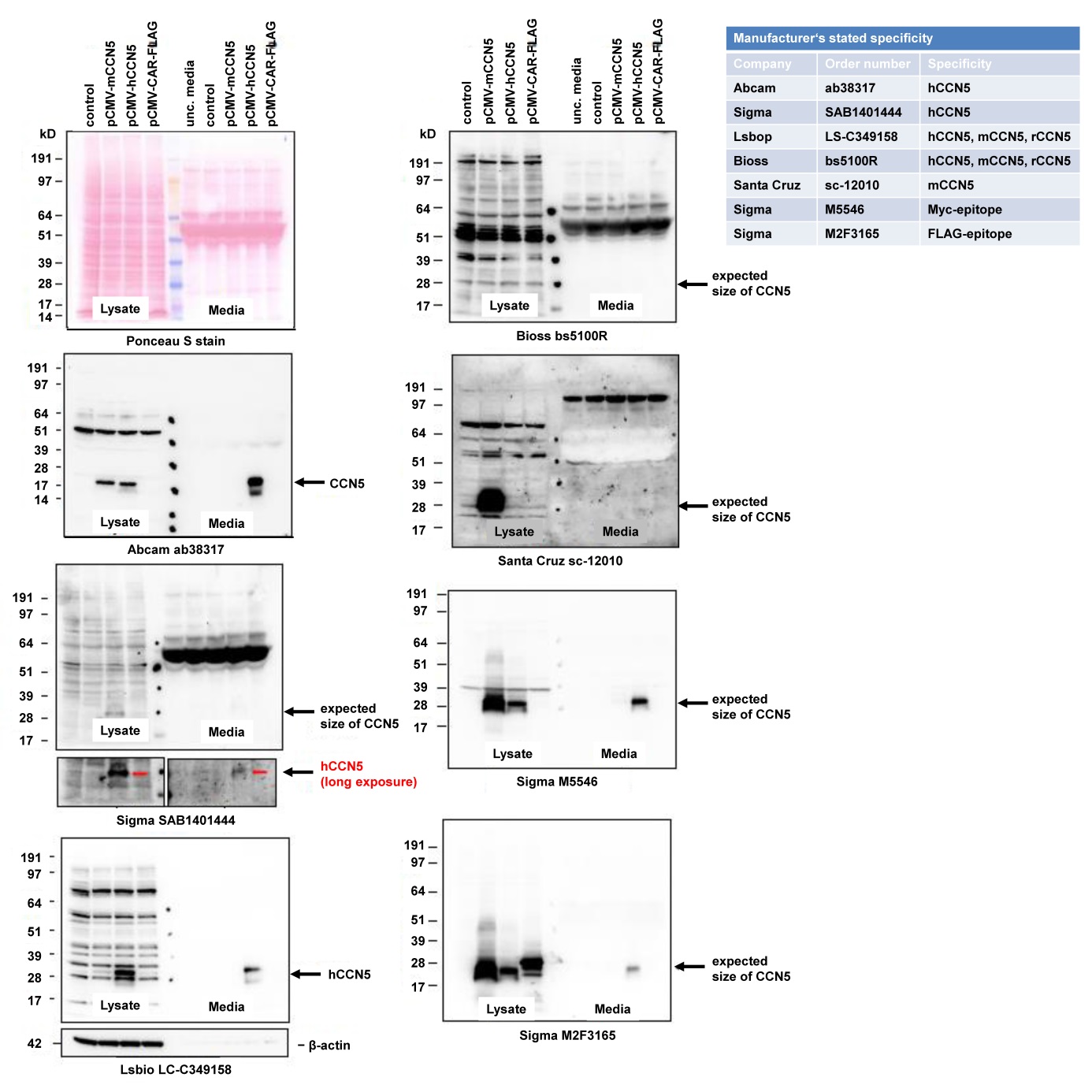


# Suppl. Figure 2: Testing for antibody specificity. HEK293 cells were either transfected with pCMV-mCCN5, pCMV-hCCN5, or pCMV-CAR-FLAG. Mock-transfected cells served as controls. Cell extracts and supernatants were harvested after 48 hours and probed with antibodies to be specific for hCCN5 (Abcam, #ab38317; Sigma, #SAB1401444), hCCN5/mCCN5/rCCN5 (LSbio, #LS-C349158; Bioss, bs #5100R), mCCN5 (Santa Cruz, #sc-12010), FLAG-epitope (Sigma, #M5546), or the myc-epitope (Sigma, #M2F3165). A representative Ponceau S stain and probing with an antibody specific for β-actin served as controls to demonstrate equal protein loading. Please note that in our hands, the antibody from Abcam that should be specific for hCCN5 also recognizes CCN5 from rodents, most likely because of the high homology of the antigen used for immunization.

#
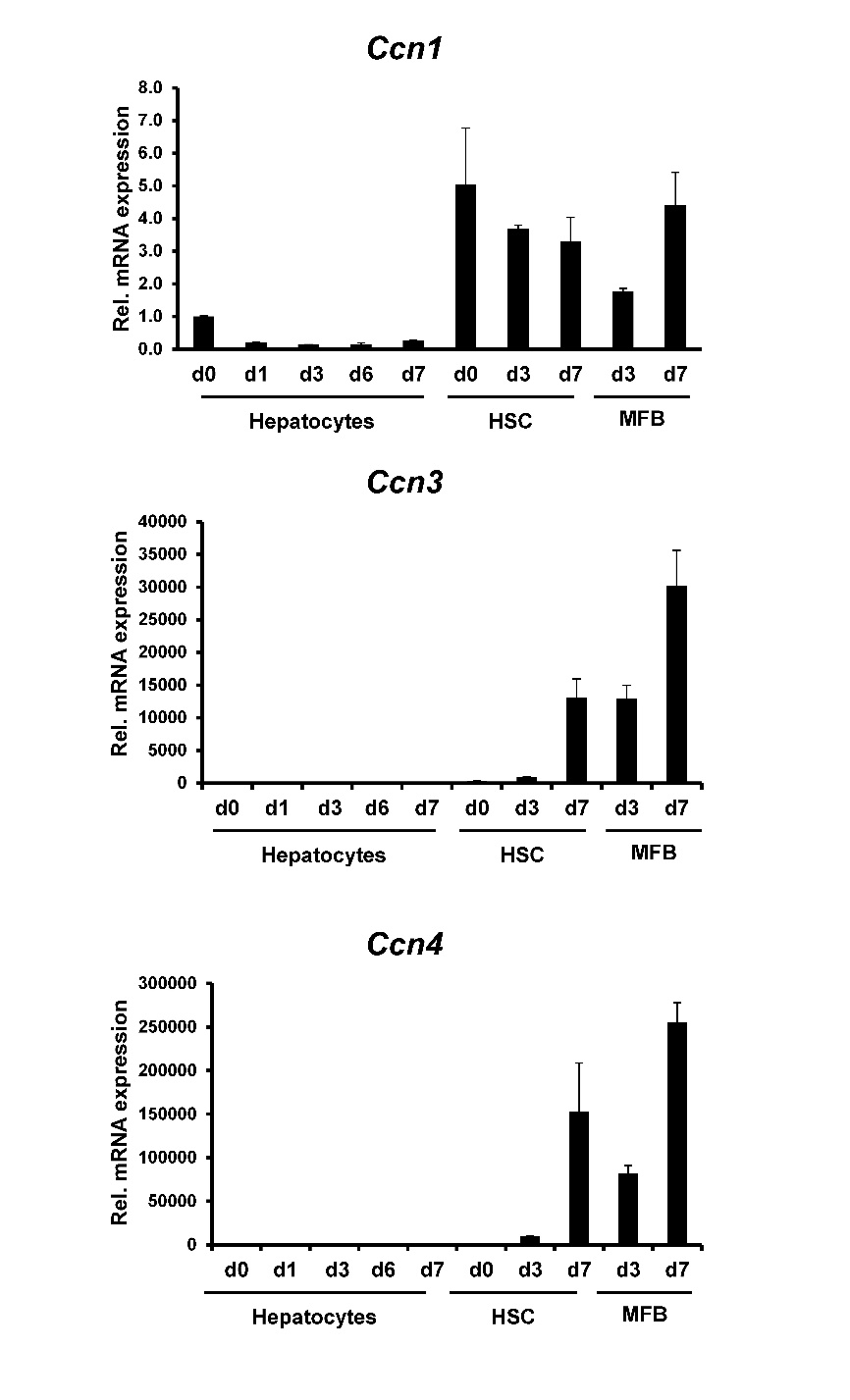


# Suppl. Figure 3: Expression of *Ccn1*, *Ccn3*, and *Ccn4* in liver cells. Primary hepatocytes, hepatic stellate cells (HSC) and myofibroblasts (MFB) derived thereof were cultured for indicated times. Total RNA was isolated and expression of *Ccn1*, *Ccn3* and *Ccn4* was analyzed by RT-qPCR.

#
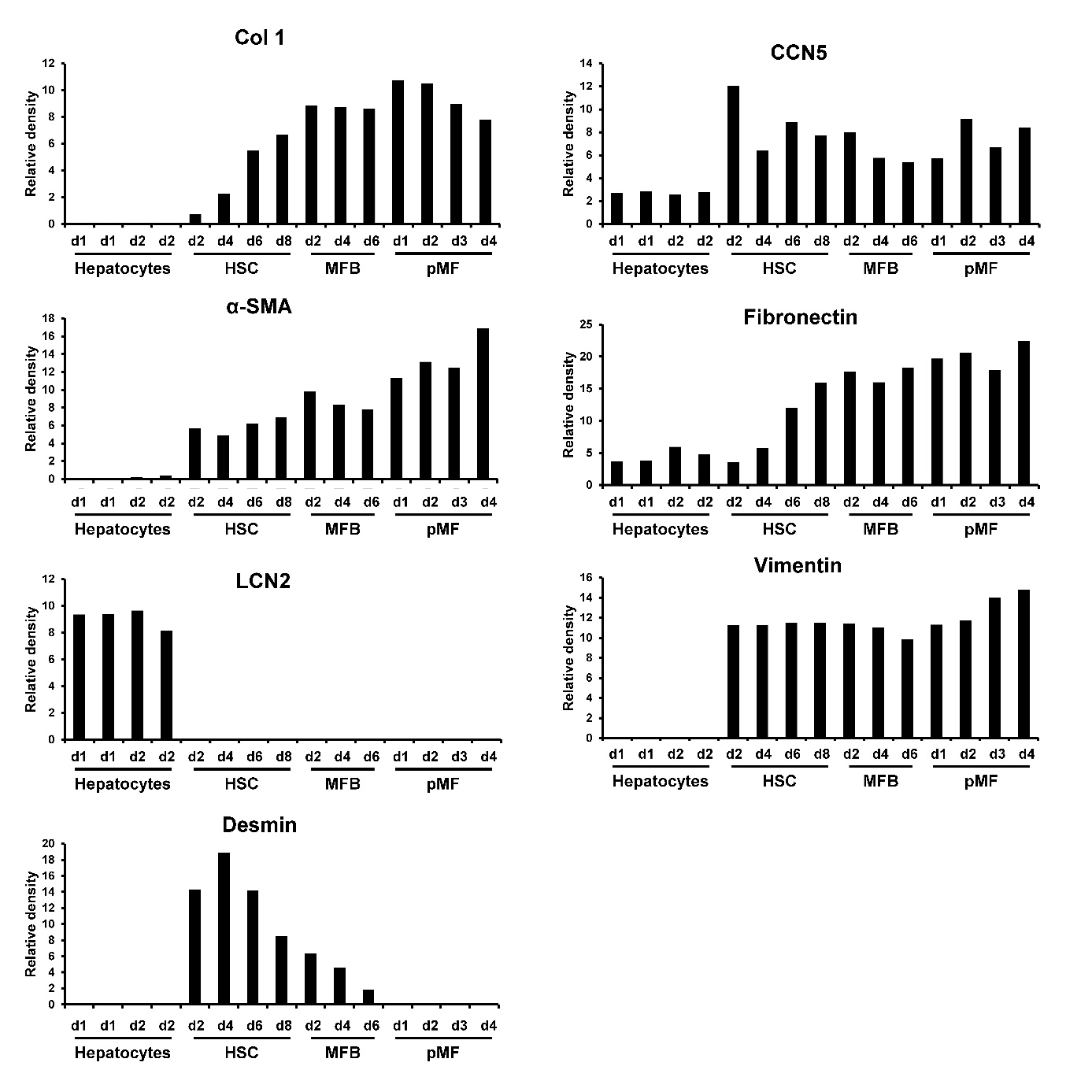


# Suppl. Figure 4: Densitometry of Western blot results depicted in Figure 1B.

#
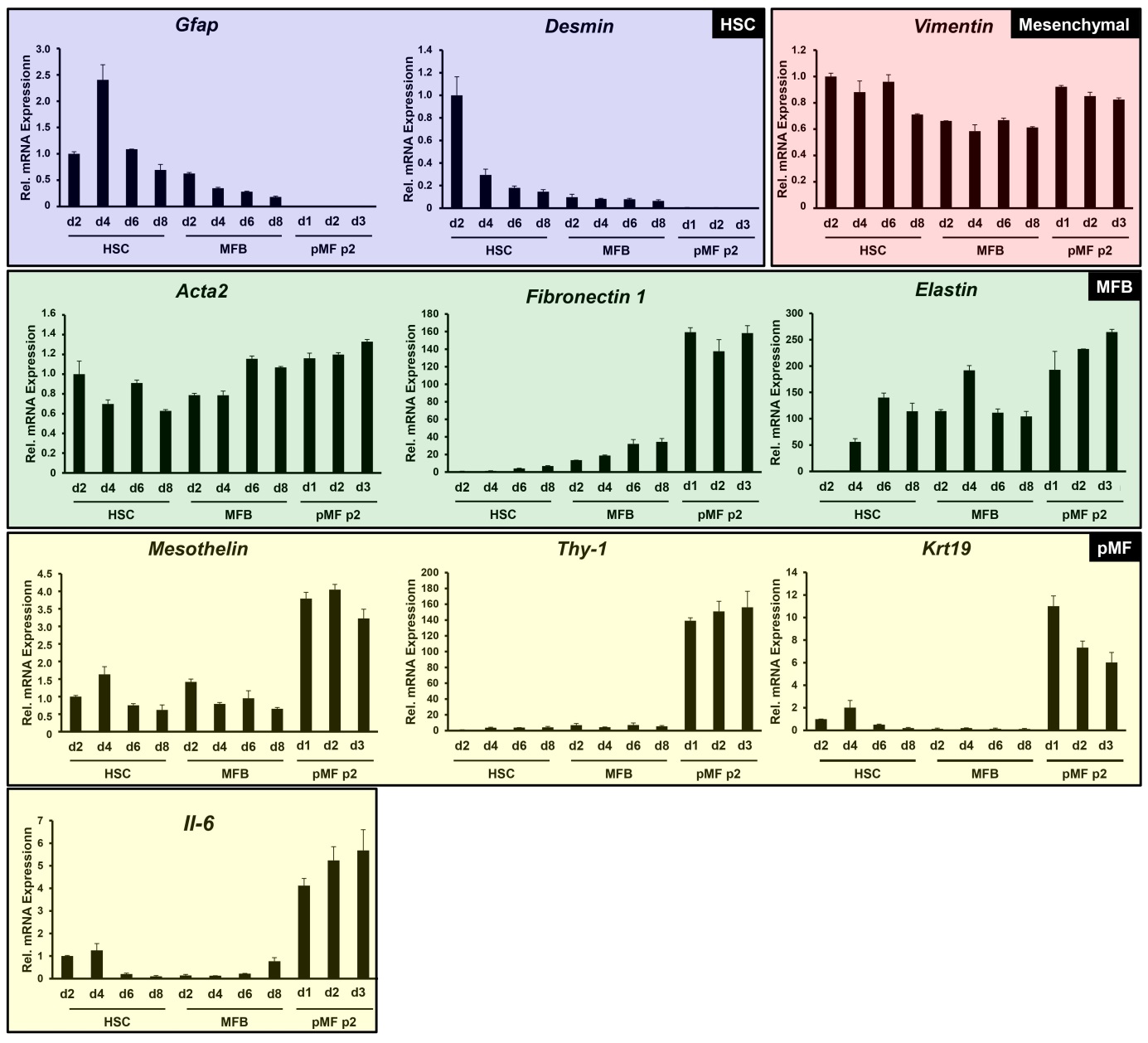


**Suppl. Figure 5: Gene expression analysis in hepatic stellate cells (HSC), myofibroblasts (MFB) and portal myofibroblasts (pMF) at passage 2 (p2).** The different primary liver cells were cultured for indicated time intervals. mRNA was isolated and the expression of glial fibrillary acidic protein (*Gfap*), Desmin (*Des*), Vimentin (*Vim*), α-smooth mucle actin (*Acta2*), Fibronectin 1 (*Fn1*), Elastin (*Eln*), Mesothelin (*Msln*), Thy-1 cell surface antigen (*Thy1*), Keratin 19 (*Krt19*) and interleukin-6 (*Il-6*) analyzed by RT-qPCR. These markers are primarily specific for HSC (boxed in *blue*), MFB (boxed in *green*), mesenchymal cells (boxed in *red*), or pMF (boxed in *yellow*).

#
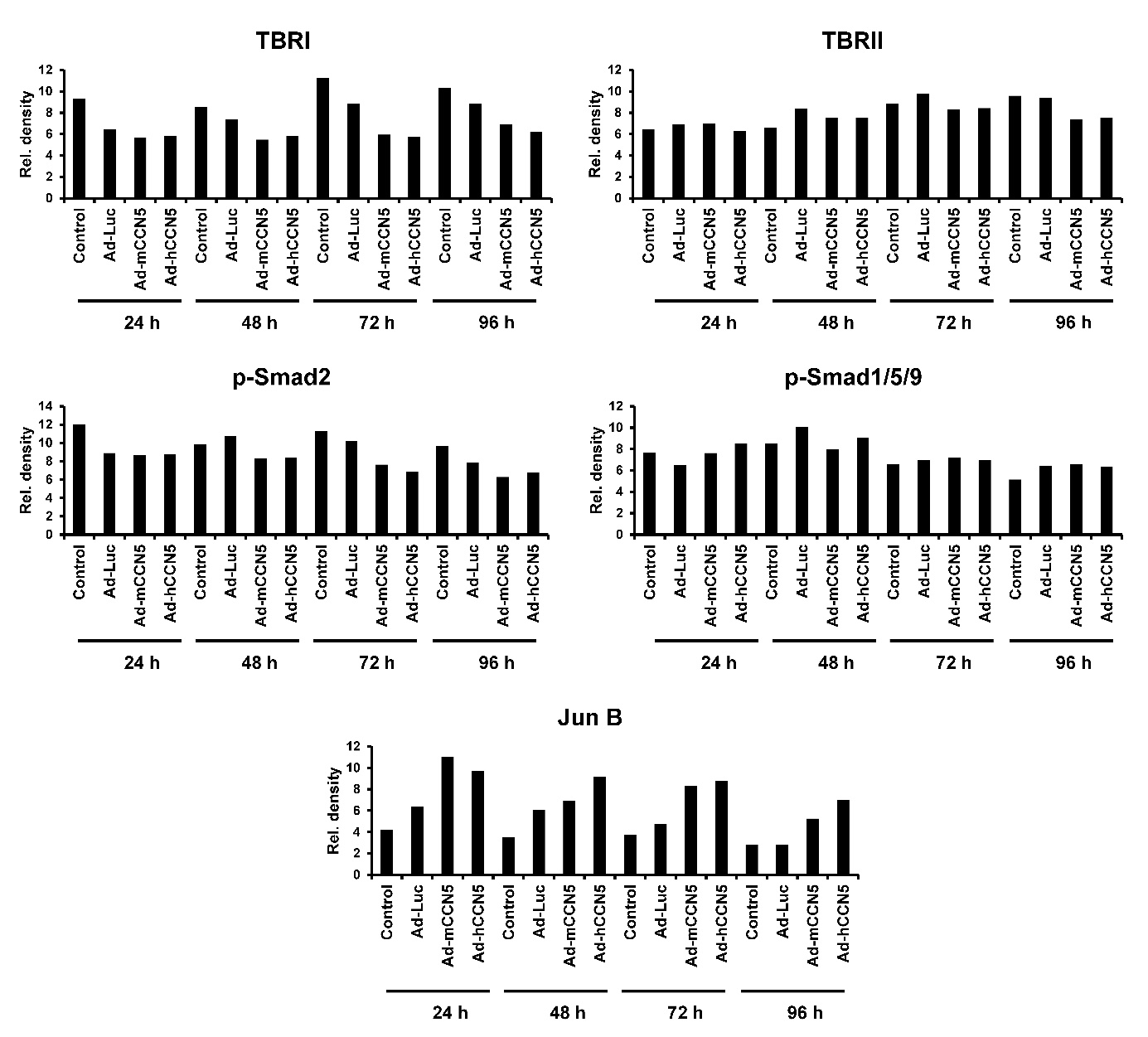


# Suppl. Figure 6: Densitometric analysis of Western blot data depicted in Figure 3C.

#
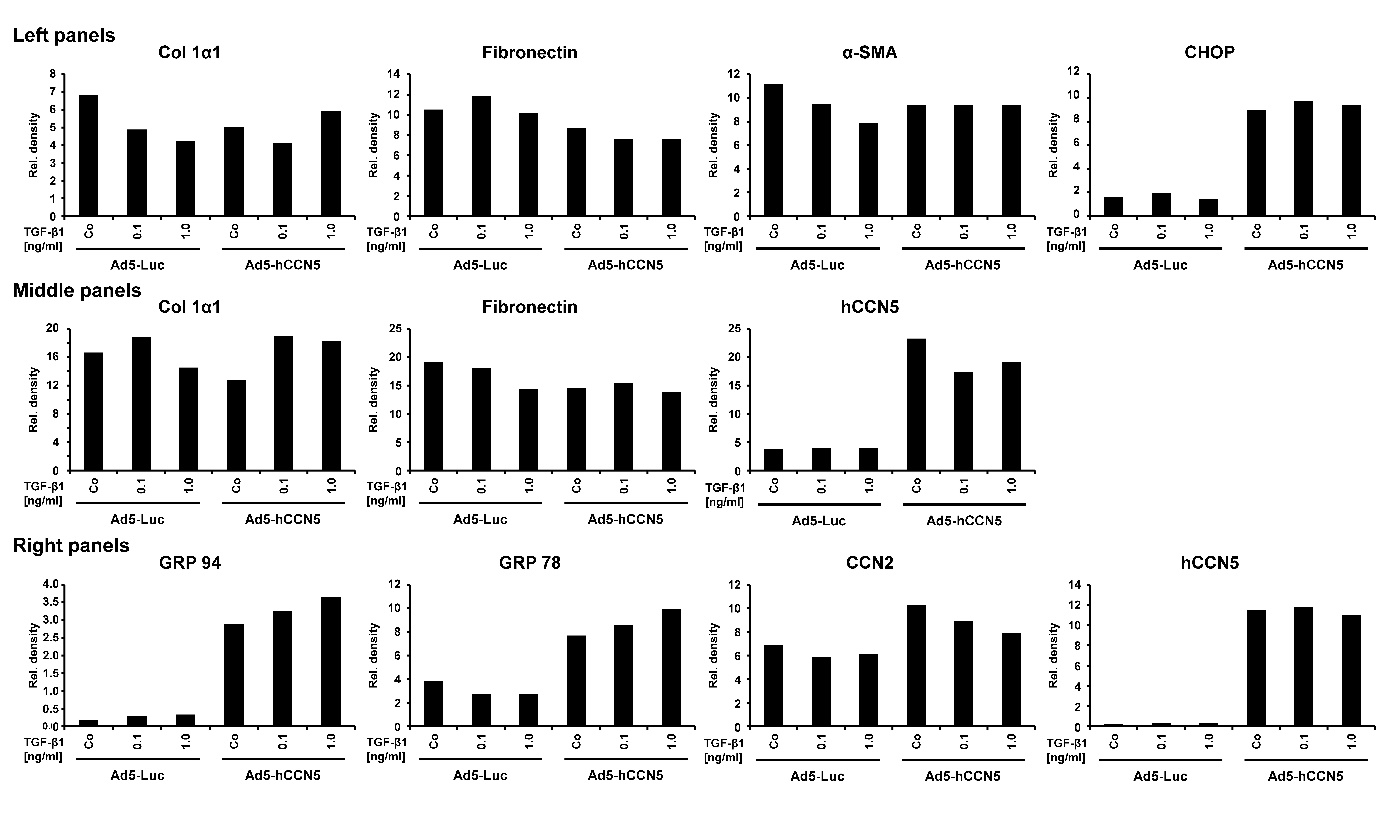


# Suppl. Figure 7: Densitometric analysis of Western blot data depicted in Figure 4.

#
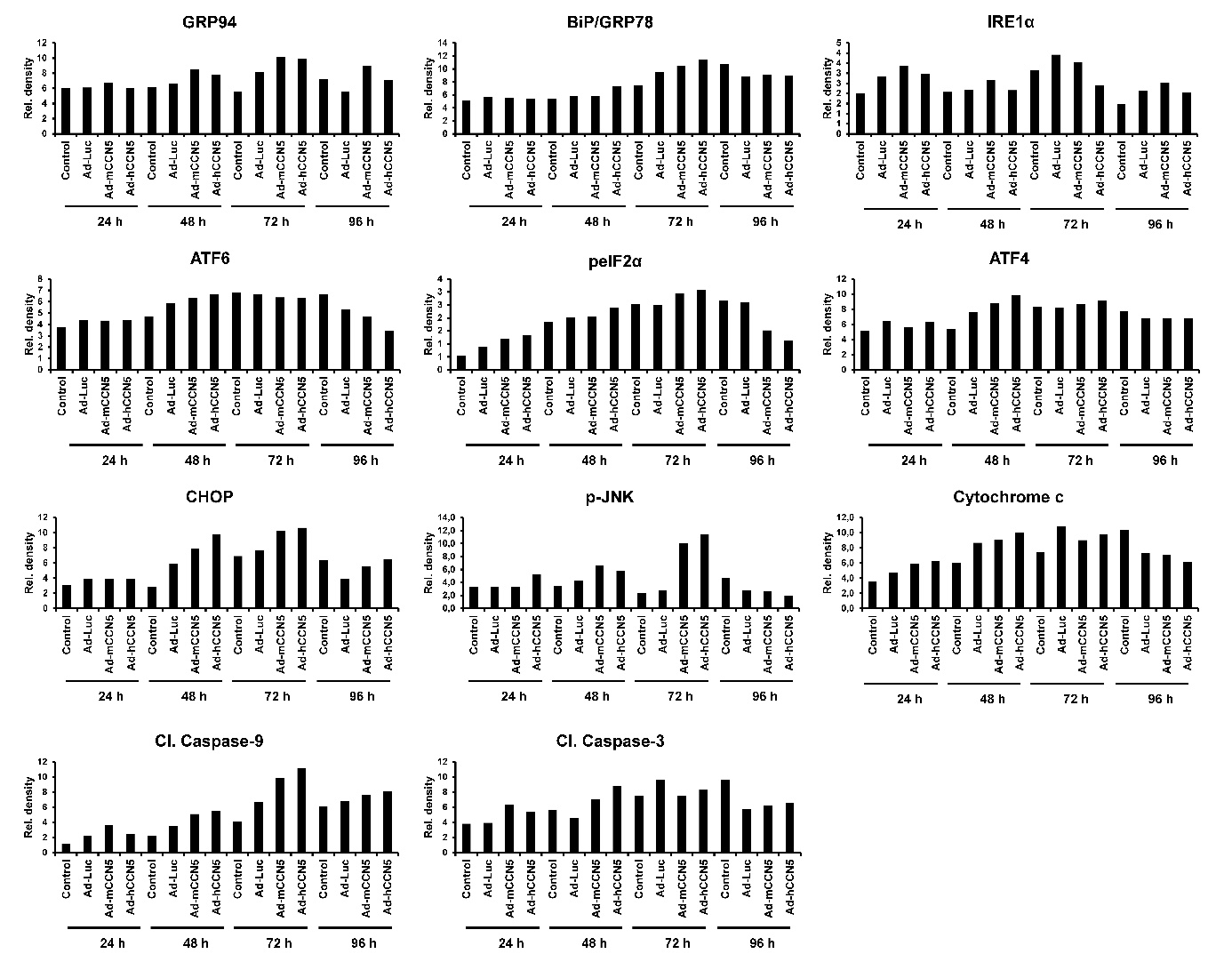


# Suppl. Figure 8: Densitometric analysis of Western blot data depicted in Figure 5C.

# Suppl. Table 1: Primer used in this study

| Gene | Acc.no. | Forward | Reverse |
| --- | --- | --- | --- |
| *rActa2* | NM_031004 | 5’-GAGGAGCATCCGACCTTGC-3’ | 5’-ATTTTCTCCCGGTTGGCC-3’ |
| *hCcn1* | NM_001554 | 5’-AAGAAACCCGGATTTGTGAG-3’ | 5’-GCTGCATTTCTTGCCCTTT-3’ |
| *mCcn1* | NM_010516 | 5’-GGATCTGTGAAGTGCGTCCT-3’ | 5’-CTGCATTTCTTGCCCTTTTT-3’ |
| *rCcn2* | NM_022266 | 5’-GCTGACCTAGAGGAAAACATTAAGA-3’ | 5’-CCGGTAGGTCTTCACATGG-3’ |
| *rCcn3* | NM_030868 | 5’-CGGCCTTGTGAGCAAGAG-3’ | 5’-TTCTTGGTCCGGAGACACTT-3’ |
| *rCcn4* | NM_031716 | 5’-ACATCCGACCACACATCAAG-3’ | 5’-AAGTTCGTGGCCTCCTCTG-3’ |
| *rCcn5* | NM_031590 | 5’-CAGGGCCTGGTTTGTCAG-3’ | 5’-CCGTCATCCTCATCCAAGA-3’ |
| *rChop* | NM_001109986 | 5’-ACCACCACACCTGAAAGC-3’ | 5’-AGCTGGACACTGTCTCAAAGG-3’ |
| *rCol Iα* | NM_053304 | 5’-CATGTTCAGCTTTGTGGACCT-3’ | 5’-GCAGCTGACTTCAGGGATGT-3’ |
| *rDesmin* | [NM_022531.2](https://www.ncbi.nlm.nih.gov/nucleotide/NM_022531.2?report=genbank&log$=nucltop&blast_rank=3&RID=TDP04ABK016) | 5’-GATCAACCTTCCGATCCAGA-3’ | 5’-TTGCTCAGGGCTGGTTTCT-3’ |
| *rElastin* | [NM_012722.1](https://www.ncbi.nlm.nih.gov/nucleotide/NM_012722.1?report=genbank&log$=nuclalign&blast_rank=31&RID=TDP2KRFA016) | 5’-GCTGATCCTCTTGCTCAACC-3’ | 5’-CTCCACCAGGCACAGCTC-3’ |
| *rFibronectin 1* | NM_019143.2 | 5’-CAGCCCCTGATTGGAGTC-3’ | 5’-TGGGTGACACCTGAGTGAAC-3’ |
| *rGapdh* | NM_017008 | 5’-TGTGAACGGATTTGGCCGTA-3’ | 5’-GATGGTGATGGGTTTCCCGT-3’ |
| *rGfap* | NM_017009.2 | 5’-TTTCTCCAACCTCCAGATCC -3’ | 5’-TCTTGAGGTGGCCTTCTGAC-3’ |
| *rGrp78/Bip* | NM_013083 | 5’-CCGTAACAATCAAGGTCTACGA-3’ | 5’-AAGGTGACTTCAATCTGGGGTA-3’ |
| *rGrp94* | NM_001012197 | 5’-GCACCATGAGGGTCCTGT-3’ | 5’-CATCGTCAGCTCTCACAAACC-3’ |
| *rIl-6* | [NM_012589.2](https://www.ncbi.nlm.nih.gov/nucleotide/NM_012589.2?report=genbank&log$=nucltop&blast_rank=1&RID=TDPB524G01N) | 5’-CCCTTCAGGAACAGCTATGAA-3’ | 5’-ACAACATCAGTCCCAAGAAGG-3’ |
| *rKrt19* | NM_199498 | 5’-CGACCTGGAGATGCAGATAGA-3’ | 5’-CCTCAGGGCACTAATTTCCTC-3’ |
| *rLcn2* | NM_130741 | 5’-CACTTCCATCCTCGTCAGG-3’ | 5’-AATATTCCCCAGGGTGAACTG-3’ |
| *rMesothelin* | [NM_031658.1](https://www.ncbi.nlm.nih.gov/nucleotide/NM_031658.1?report=genbank&log$=nucltop&blast_rank=6&RID=TDPJPEXN01N) | 5’-TGCAGACCCAGACTACAAGGA-3’ | 5’-AAGAGGCCTGTGGGAAGACT-3’ |
| *rTgfb1* | NM_021578 | 5’-CCTGGAAAGGGCTCAACAC-3’ | 5’-CAGTTCTTCTCTGTGGAGCTGA-3’ |
| *rThy-1* | NM_012673 | 5’-CCACAAGCTCCAATAAAACTATCAA-3’ | 5’-AGCAGCCAGGAAGTGTTTTG-3’ |
| *rVimentin* | [NM_031140.1](https://www.ncbi.nlm.nih.gov/nucleotide/NM_031140.1?report=genbank&log$=nucltop&blast_rank=6&RID=TDPFG2W301N) | 5’-CGAGAAAAATTGCAGGAGGA-3’ | 5’-GAATGACTGCAGGGTGCTCT-3’ |
| *rXbp1** | NM_001004210 | 5’-TTACGAGAGAAAACTCATGGGC-3’ | 5’-GGGTCCAACTTGTCCAGAATGC-3’ |

# * sizes of amplicons expected are: unspliced Xbp1 (Xbp1 (u))= 289 bp, spliced Xbp1(Xbp1 (s)) = 263 bp

# Suppl. Table 2: Sources of primary antibodies used in this study

| Protein | Antibody | Source | Clonality | Epitope | Specificity* | Dilution |
| --- | --- | --- | --- | --- | --- | --- |
| α-SMA | CBL171 | Cymbus Biotech, Hampshire, UK | Monoclonal (mouse) | Synthetic peptide corresponding to the ten *N*-terminal amino acids of the α-smooth muscle isoform of actin | b, ch, eq, h, m, r | 1:2,000 |
| β-actin | A5441 | Sigma, Taufkirchen, Germany | Monoclonal (mouse) | Sightly modified β-cytoplasmic actin *N*-terminal peptide (Ac-Asp-Asp-Asp-Ile-Ala-Ala-Leu-Val-Ile-Asp-Asn-Gly-Ser-Gly-Lys) conjugated to KLH | sh, c, fe, ch, r, m, hm, ra, ca, p, h, b, gp | 1:10,000 |
| ATF4 | sc-390063 | Santa Cruz, Santa Cruz, CA, USA | Monoclonal (mouse) | Amino acids 1-290 of human ATF4 | h, m, r | 1:1,000 |
| ATF6α | sc-166659 | Santa Cruz | Monoclonal (mouse) | Amino acids 31-310 of human ATF6 | h, m, r | 1:1,000 |
| CCN2/CTGF | sc-14939 | Santa Cruz | Polyclonal (goat) | Peptide mapping within an internal region of human CTGF/CCN2 | h, m, r | 1:1,000 |
| CCN5 | ab38317 | Abcam, Cambridge, UK | Polyclonal (rat) | A KLH-conjugated synthetic peptide (10-30 aa in length) in the region of 127~141 of human CCN5 | h | 1:1,000 |
| CCN5 | SAB1401444 | Sigma | Monoclonal (mouse) | GST-tagged full-length human CCN5 (aa24-aa250) | h | 1:1,000 |
| CCN5 | LS-C349158 | LSBio, Seattle, CA, USA | Polyclonal (rabbit) | Recombinant fusion protein containing a sequence corresponding to amino acids 1-250 of human CCN5 | h, m, r | 1:1,000 |
| CCN5 | bs-5100R | Bioss, Biozol, Eching, Germany | Polyclonal (rabbit) | KLH conjugated synthetic peptide derived from human CCN5 | h, m, r | 1:1,000 |
| CCN5 | sc-12010 | Santa Cruz | Polyclonal (goat) | Peptide mapping within an internal region of CCN5 of mouse origin | NN | 1:1,000 |
| CHOP | 5554 | Cell Signaling, Frankfurt am Main, Germany | Monoclonal (rabbit) | Synthetic peptide corresponding to residues surrounding Leu159 of human CHOP protein | h, r | 1:1,000 |
| cleaved Caspase-3 | 9664 | Cell Signaling | Monoclonal (rabbit) | Synthetic peptide corresponding to amino-terminal residues adjacent to Asp175 of human caspase-3 | h, m, r, mk, b, d, p | 1:1,000 |
| cleaved Caspase-9 | 9507 | Cell Signaling | Polyclonal (rabbit) | Synthetic peptide corresponding to amino-terminal residues surrounding Asp353 of rat caspase-9 | m, r | 1:1,000 |
| Collagen 1 | PS065 | Monosan, Uden, The Netherlands | Polyclonal (rabbit) | Native collagen type I from rat tail tendon | rt | 1:500 |
| Cytochrome c | 11940 | Cell Signaling | Monoclonal (rabbit) | Synthetic peptide corresponding to residues surrounding Pro72 of human cytochrome c protein | h, m ,r, mk | 1:1,000 |
| Desmin | NB110-56931 | Novus Biologicals LLC, R&D Systems, Wiesbaden-Nordenstadt, Germany | Monoclonal (rabbit) | Synthetic peptide corresponding to the *C*-terminus of human desmin | h, m. r, gp | 1:1,000 |
| eIF2α | 9722 | Cell Signaling | Polyclonal (rabbit) | Synthetic peptide corresponding to the carboxy-terminal sequence of eIF2α | h, m, r, mk | 1:1,000 |
| Fibronectin | AB1954 | Millipore, Sigma-Aldrich, Taufkirchen, Germany | Polyclonal (rabbit) | Rat plasma fibronectin | h, m, r | 1:2,000 |
| FLAG-epitope | F3165 | Sigma | Monoclonal (mouse) | Synthetic peptide (DYKDDDDK) | FLAG sequence | 1:5,000 |
| GAPDH | sc-32233 | Santa Cruz | Monoclonal (mouse) | GAPDH purified from rat muscle | h, m, r | 1:1,000 |
| GRP78/BIP | 3177 | Cell Signaling | Monoclonal (rabbit) | Synthetic peptide corresponding to residues surrounding Gly584 of human BiP | h, m | 1:1,000 |
| GRP94 | sc-393402 | Santa Cruz | Monoclonal (mouse) | Amino acids 200-411 of human GRP94 | h, m, r | 1:1,000 |
| HSP 70 | sc-24 | Santa Cruz | Monoclonal (mouse) | Human HSP 70 isolated from HeLa cells | h, m, r | 1:500 |
| IRE1α | 3294 | Cell Signaling | Monoclonal (rabbit) | Synthetic peptide corresponding to residues surrounding His963 of human IRE1α | h, m, r | 1:1,000 |
| Jun B | 3753 | Cell Signaling | Monoclonal (rabbit) | Synthetic peptide corresponding to residues surrounding Pro169 of human JunB | h, m, r | 1:1,000 |
| JNK2 | 9258 | Cell Signaling | Monoclonal (rabbit) | Human JNK2/MBP fusion protein | h, m, r, ha, mk, mi | 1:1,000 |
| LCN2 | AF3508 | R&D Systems | Polyclonal (goat) | Mouse myeloma cell line NS0-derived recombinant rat Lipocalin-2/NGAL Gln21-Asn198 | m | 1:1,000 |
| Luciferase | sc-74548 | Santa Cruz | Monoclonal (mouse) | Amino acids 251-550 mapping at the *C*-terminus of Luciferase of *Photinus pyralis* | Luciferase | 1:1,000 |
| Myc-epitope | M5546 | Sigma | Monoclonal (mouse) | Synthetic peptide of human p62 c-Myc protein | Myc-tag sequence | 1:5,000 |
| peIF2α | 3597 | Cell Signaling | Monoclonal (rabbit) | Synthetic phosphopeptide corresponding to residues surrounding Ser51 of human eIF2α | h, m, r, mk, d | 1:1,000 |
| pJNK | 4668 | Cell Signaling | Monoclonal (rabbit) | Synthetic phosphopeptide corresponding to residues surrounding Thr183/Tyr185 of human SAPK/JNK | h, m, r | 1:2,000 |
| pSmad1/5/9 | 13820 | Cell Signaling | Monoclonal (rabbit) | Synthetic phosphopeptide corresponding to residues surrounding Ser463/465 of human Smad1 and Smad5 proteins | h, m, r | 1:1,000 |
| pSmad2 | 8828 | Cell Signaling | Monoclonal (rabbit) | Synthetic peptide corresponding to residues surrounding Ser465/467 of human Smad2 | h, m, r, mk | 1:1,000 |
| Smad1 | 9743 | Cell Signaling | Polyclonal (rabbit) | Synthetic peptide corresponding to residues surrounding Ser190 of human Smad1 | h, m, mk | 1:1,000 |
| Smad2 | 3103 | Cell Signaling | Monoclonal (mouse) | Recombinant protein specific to the amino terminus of human Smad2 protein | h, m, r, mk | 1:1,000 |
| TGFβRI | sc-398 | Santa Cruz | Polyclonal (rabbit) | Peptide mapping within the cytoplasmic domain of human TGFβRI | h, m, r, o | 1:1,000 |
| TGFβII | sc-400 | Santa Cruz | Polyclonal (rabbit) | Peptide mapping within the cytoplasmic domain of human TGFβRII | h, m, r | 1:1,000 |
| Vimentin | ab92547 | Abcam | Monoclonal (rabbit) | Unspecified synthetic peptide within human Vimentin aa400 to the *C*-terminus | h, m, r, agm, ct, p, rmk | 1:2,000 |

# Abbreviations used are: agm = African green monkey; b = bovine; c = carp; ca = canine; ch = chicken; ct = cat; d = dog; eq = horse; fe = feline; gp= guinea pig; h = human, ha = hamster, hm = *Hirudo medicinalis*; m = mouse, mi = mink, mk = monkey, o = ovine; p = pig; r = rat, ra = rabbit; rmk = Rhesus monkey; sh = sheep.
